# Add, subtract and multiply: Meta-analyses of brain correlates of arithmetic operations in children and adults

**DOI:** 10.1016/j.dcn.2024.101419

**Published:** 2024-07-26

**Authors:** Asya Istomina, Marie Arsalidou

**Affiliations:** a HSE University, the Russian Federation; b NeuroPsyLab, Canada; c York University, Toronto, Canada

**Keywords:** Meta-analysis, Arithmetic, Mathematics, Calculation, Children, Adults, Fronto-parietal network, Cingulo-opercular network

## Abstract

Mathematical operations are cognitive actions we take to calculate relations among numbers. Arithmetic operations, addition, subtraction, multiplication, and division are elemental in education. Addition is the first one taught in school and is most popular in functional magnetic resonance imaging (fMRI) studies. Division, typically taught last is least studied with fMRI. fMRI meta-analyses show that arithmetic operations activate brain areas in parietal, cingulate and insular cortices for children and adults. Critically, no meta-analysis examines concordance across brain correlates of separate arithmetic operations in children and adults. We review and examine using quantitative meta-analyses data from fMRI articles that report brain coordinates separately for addition, subtraction, multiplication, and division in children and adults. Results show that arithmetic operations elicit common areas of concordance in fronto-parietal and cingulo-opercular networks in adults and children. Between operations differences are observed primarily for adults. Interestingly, higher within-group concordance, expressed in activation likelihood estimates, is found in brain areas associated with the cingulo-opercular network rather than the fronto-parietal network in children, areas also common between adults and children. Findings are discussed in relation to constructivist cognitive theory and practical directions for future research.

## 1 Introduction

Children typically start elementary school with a basic understanding of numbers. In the next four years of schooling, they learn to add, subtract, multiply and divide, usually in that order. Many behavioral studies examine the mechanisms of learning mathematical operations (e.g., Lemaire and Siegler, 1995; Barrouillet et al., 2008), as they form the foundations for future academic performance (Shalev et al., 2005, Watts et al., 2014). Mathematical operations encompass a broader range of operations beyond basic arithmetic. Basic arithmetic operations involve addition, subtraction, multiplication, and division. Many functional Magnetic Resonance Imaging (fMRI) studies examine the brain correlates of mathematical processes such as numerical cognition in adults (Arsalidou and Taylor, 2011, Pollack and Ashby, 2018, Hawes et al., 2019, Sokolowski et al., 2023 for meta-analyses) and children (Pollack and Ashby, 2018, Arsalidou et al., 2018 for meta-analyses). Some of these meta-analyses examine the brain correlates of arithmetic operations in adults, identifying concordance in prefrontal, parietal, cingulate and insular cortices (Arsalidou and Taylor, 2011, Pollack and Ashby, 2018, Hawes et al., 2019, Sokolowski et al., 2023). Meta-analyses of arithmetic operations in children show activation in parietal, cingulate and insular cortices, but not prefrontal cortices (Arsalidou et al., 2018, Pollack and Ashby, 2018). Critically, no review or meta-analysis to date examines concordance of separate arithmetic operations in children. As different arithmetic operations are said to rely on different strategies (e.g., addition and multiplication on retrieval, and subtraction on procedural; Grabner and De Smedt, 2011), it may be reasonable to expect that arithmetic operations may have some differences in brain representation. However, developmental cognitive theory suggest that learning relies on effortful attentional processes that can be applied during problem solving regardless of operations, and brain representation should be comparable when attentional requirements are comparable (Pascual-Leone, 1970, Agostino et al., 2010; Pascual-Leone et al., 2010). The current paper systematically reviews the adult and children fMRI literature on arithmetic operations and maps concordant brain areas across eligible articles using quantitative meta-analyses.

The four adult-focused meta-analyses generally reveal a set of concordant regions in parietal, prefrontal, cingulate and insular cortices (Arsalidou and Taylor, 2011, Pollack and Ashby, 2018, Hawes et al., 2019, Sokolowski et al., 2023). Children-focused fMRI work is less extensive. A review by Peters and De Smedt (2018) proposes that mathematical cognition in children implicates parietal, and temporal brain areas. Some suggest that prefrontal cortical activity exhibits a comparatively reduced magnitude when directly contrasted with that of adults, as highlighted in previous studies (Davis et al., 2009, Kucian et al., 2008). Notably, two quantitively meta-analyses that explore fMRI data from studies that examined brain responses to arithmetic operations in children identify cingulate, insular and parietal cortices, but not prefrontal and temporal regions (Pollack and Ashby, 2018, Arsalidou et al., 2018). Specifically, Pollack and Ashby report concordance in the left superior frontal gyrus (Brodmann area (BA 6), right medial frontal gyrus (BA 8), cingulate gyrus (BA 32), and the right insula (BA 13). Arsalidou et al. (2018), in addition to the aforementioned areas in cingulate and insular cortices, report concordance in the parietal cortex. Specifically, left precuneus (BA 19) and inferior parietal lobule (BA 40), encompassing parts of the inferior parietal sulcus and angular gyrus (BA 39), alongside the right precuneus (BA 7).

One early adult meta-analysis examined separately concordance of addition, subtraction and multiplication (Arsalidou and Taylor, 2011). Specifically, addition tasks elicit concordant activation in visual areas, parietal regions, frontal and prefrontal cortices, bilateral thalamus, right insula (BA 13), right claustrum, and bilateral cerebellum. Similarly, solving subtraction problems is linked to occipito-temporal visual regions, parietal areas, frontal and prefrontal regions, with additional activations observed in bilateral insula (BA 13) and right cerebellum. For multiplication tasks, activity is observed in occipito-temporal visual regions, parietal areas, temporal regions, frontal and prefrontal regions. Furthermore, consistent activations are seen in bilateral cingulate gyri (BA 32), bilateral thalami, left claustrum, right insula, right caudate body, and right cerebellum across different studies.

This current report will be the first to examine concordance of separate arithmetic operations in children in comparison with adults, as the question remains whether the same brain regions are involved in solving tasks related to fundamental arithmetic operations in children and adults. Theoretically, it is expected that concordance in children will favor the insula and cingulate related to affective factors such as motivation and to a lesser extent the cognitive network that includes prefrontal and parietal regions (Arsalidou and Pascual-Leone, 2016). We do not anticipate differences among arithmetic operations.

## 2 Method

### 2.1 Literature review

**Fig. 1:**
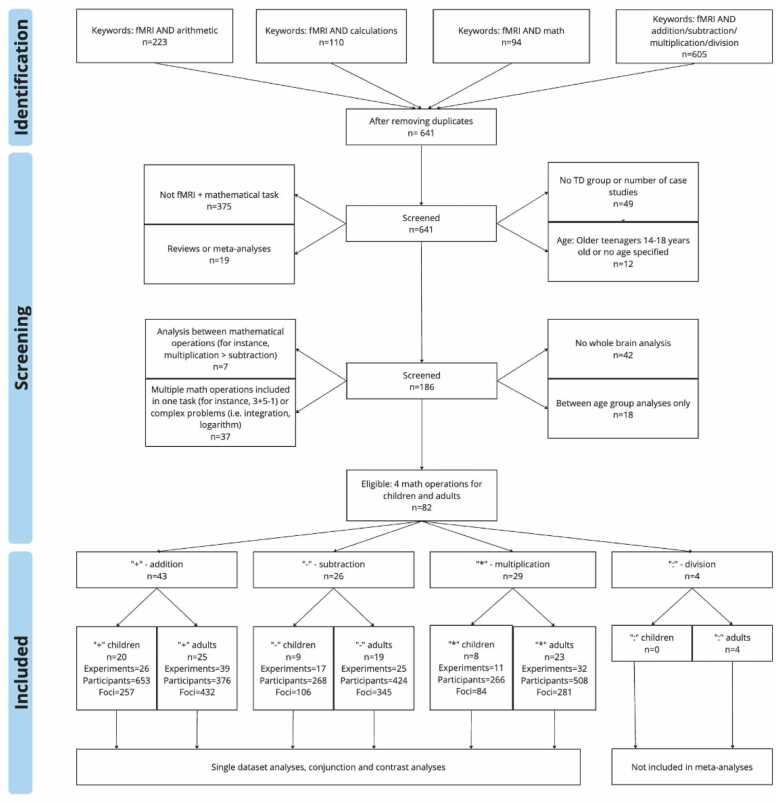
PRISMA flowchart showing the stages of searching for articles for this study (template by Page et al., 2021), n = number of papers, TD =typically developing.

PRISMA guidelines were used to document the steps taken for the systematic review and meta-analyses (Page et al., 2021). PubMed was searched using terms: fMRI and arithmetic, fMRI and calculations, fMRI and math, fMRI and addition/subtraction/multiplication/division in June 2023, searching all previous years. The search yielded a total of 1032, which resulted into 641 after duplicates were removed. Details for searches and steps taken for article eligibility are illustrated on Fig. 1.

### 2.2 Article eligibility criteria

Articles were subjected to two sets of inclusion/exclusion criteria. The initial stage of the screening process defined eligible the articles that (a) use fMRI, (b) present whole-brain group analyses accompanied by stereotactic coordinates expressed in either Talairach/Tournoux or Montreal Neurological Institute (MNI) space, (c) employ a minimum of one of the subsequent tasks: addition, subtraction, multiplication, division, (d) publish in the English language, (e) present number stimuli to participants in digits, words or had a symbolic designation, and (f) include a group of healthy children (up to mean age of 14 years) or adults (> 18 years). The second stage involved identifying eligible contrasts, by considering arithmetic operation used, types of statistical analyses conducted, and eligible contrasts (i.e., experiments). Eligible experiments were subtraction contrasts (e.g., task > control) rather than correlational analyses or multivariate analyses.

Contrasts (i.e., experiments) were systematically categorized according to the specific category of arithmetic operation for children (Table 1) and for adults (Table 2). Articles that reported results of mixed arithmetic operations (e.g., 3+2–4) or other complex mathematical operations (e.g., integration), were not considered. Inclusion criteria for addition, subtraction, and multiplication maps (division did not yield sufficient experiments: Fig. 1) were meticulously outlined as follows. Although we did not employ a behavioral performance criterion, we did verify that fMRI contrasts that involve tasks of different difficulty were expressed also in behavioral performance. That is, to include contrast Math task Hard > Math task Easy in the corresponding map, it was necessary to ensure that task Math task Easy was easier than task Math task Hard by way of differences expressed in behavioral scores, such as reaction time, accuracy, or performance efficiency.

**Table 1:** List of eligible articles with children.

	Author	Year	Sample				Contrast	Contrast type: (ADD, SUB, MUL)
			N	F	Hand	Age		
1.	Kawashima	2004	8	4	R	Range 9 – 14	Addition > Baseline control	ADD
						Mean 11.6	Subtraction > Baseline control	SUB
							Multiplication > Baseline control	MUL
2.	Davis	2009b	27	14	n/r	Range 7.1 – 9.4	Single-digit exact calculation > Control task	ADD
						Mean 8.1	Double-digit exact calculation > Control task	ADD
							Single Digit Approximation > Control task	ADD
							Double Digit Approximation > Control task	ADD
3.	Davis	2009b	24	12	n/r	Range 8.1 – 9.1	Exact calculation task > Control task (Greek letter matching)	ADD
						Mean 8.2	Approximate calculation task Control task (Greek letter matching)	ADD
4.	Meintjes	2010	16	n/r	R	Range 8 – 12	Exact addition > Control task	ADD
						Mean n/r		
5.	Rosenberg-Lee	2011	90	39	R	Range 7.03 – 8.4 and 7.9 – 9.4	Complex addition > Simple addition	ADD
						Mean 7.67 and 8.67		
6.	Krinzinger	2011	20	9	n/r	Range 6 – 12	Symbolic exact addition > Control task	ADD
						Mean n/r	Non-symbolic exact addition > Control task	ADD
7.	Ashkenazi	2012	17	11	n/r	Range 7 – 9	Complex addition > Simple addition	ADD
						Mean 8.12		
8.	Cho	2012	86	n/r	R	Range 7 – 9	Standard addition > Control task («Plus 1» addition)	ADD
						Mean 7.7		
9.	Du	2013	19	10	n/r	Range 10.12 – 11.26	Approximate addition > Control task (Exact addition)	ADD
						Mean 10.62		
10.	Metcalfe	2013	74	40	R	Range 7 – 9	Complex addition > Control addition	ADD
						Mean 7.8		
11.	Qin	2014	28	13	n/r	Range 7 – 11	Addition > Control collapsing across Time−1	ADD
						Mean n/r	and Time−2	
12.	Prado	2014	34	21	n/r	Range 8.47 – 13.56	Large subtraction > Small subtraction	SUB
						Mean 11.54	Small subtraction > Baseline	SUB
							Large multiplication > Small multiplication	MUL
13.	Iuculano	2015	15	8	n/r	Range 7.5 – 9.6	Arithmetic problem solving before tutoring > Arithmetic problem solving after tutoring	ADD
						Mean n/r		
14.	Berteletti	2015	39	22	n/r	Range 8.5 – 13.7	Large subtraction > Small subtraction	SUB
						Mean 11.4		
15.	Demir	2015	40	23	R	Range 9 – 12	Subtraction large > Baseline	SUB
						Mean 10.9	Subtraction small > Baseline	SUB
16.	Demir-Lira	2016	33	20	n/r	Range 8 – 13	Subtraction task > Baseline	SUB
						Mean 10.9		
17.	Chang	2016	25	14	R	Range 7.7 – 10.7	Complex subtraction > Number Identification	SUB
						Mean 8.8		
18.	Peters	2016	22	10	5 L	Range 9 – 12	Subtraction Digits > Fixation	SUB
						Mean 10.73	Subtraction Words > Fixation	SUB
							Subtraction Dots > Fixation	SUB
							Subtraction Dots > Subtraction Digits	SUB
							Subtraction Dots > Subtraction Words	SUB
							Subtraction Words > Subtraction Digits	SUB
19.	Soylu	2018	24	12	n/r	Range 8.1 – 8.7	Addition problems > Baseline	ADD
						Mean 8.4	Subtraction problems > Baseline	SUB
20.	Rosenberg-Lee	2018	15	5	R	Range 8 – 9	Pre-Post training: Arithmetic verification task > Control	ADD
						Mean 8.8		
21.	Polspoel	2019	20	15	4 L	Range 9.33 – 10.76	Multiplication verification task > Jittered inter-trial interval	MUL
						Mean 9.79		
22.	Matejko	2019	42	20	2 L	Range 7.5 – 10.4	Addition task: large problem > Plus 1 Problem	ADD
						Mean 9.2	Addition task: small problem> Plus 1 Problem	ADD
23.	Wakefield	2019	20	12	R	Range 7 – 9	Mathematical equivalence problems > Rest	ADD
						Mean 8		
24.	Demir-Lira	2020	59	29	n/r	Range 9 – 12	Single-digit multiplication problems > Baseline	MUL
						Mean 11.2		
25.	Clark	2020	13	9	n/r	Range 8 – 11	Binary and decimal block > Baseline rest	ADD
						Mean 9.71		
26.	Newman	2021	43	21	n/r	Range n/r	2 groups:	
						Mean 8.3		
			22	11	n/r	Range n/r	Structured Block play group, The Blocks Rock! Game: Pre >Post training	ADD
						Mean 8.38		SUB
			21	10	n/r	Range n/r	Free Block play group, The Blocks Rock! Game: Pre > Post training	SUB
						Mean 8.29		
27.	Suárez-Pellicioni	2021	50	26	R	Range n/r	Single-digit multiplication verification task (small problems) > Control task	MUL
						Mean 11.1		
28.	Matejko	2021	38	17	2 L	Range 7.7 – 10.4	Addition large problem task > Addition small problem task	ADD
						Mean 9.2		
29.	Declercq	2022	26	13	n/r	Range n/r	Double-digit multiplication (trained) > Single-digit multiplication (pre-training session)	MUL
						Mean 10.4	Double-digit multiplication (untrained) > Single-digit multiplication (pre-training session)	MUL
							Double-digit multiplication (untrained) > Single-digit multiplication (post-training session)	MUL
							Double-digit multiplication (untrained) > Double-digit multiplication (trained)	MUL
30.	Dumontheil	2022	34	17	n/r	Range 11 – 15	Numerical Stroop Task Mixed > Numerical Stroop task Congruent	ADD
						Mean 13.4		
31.	Suárez-Pellicioni	2022	51	28	n/r	Range n/r	Single-digit multiplication verification task > Control task	MUL
						Mean 11 / 13		
32.	Amalric	2022	18	12	n/r	Range n/r	Forced-choice math task > Math naturalistic video lesson	MUL
						Mean 9.25

**Table 2:** List of eligible articles with adults.

	Author	Year	Sample				Contrast	Contrast type: (ADD, SUB, MUL)
			N	F	Hand	Age		
1.	Dehaene	1999	7	4	R	Range 22 – 28	Approximate addition > Exact addition	ADD
						Mean 25		
2.	Chochon	1999	8	4	R	Range 20 – 30	Multiplication > Digit Naming	MUL
						Mean 25	Multiplication > Comparison	MUL
							Subtraction > Digit Naming	SUB
							Subtraction > Comparison	SUB
3.	Stanescu-Cosson	2000	7	4	R	Range 22 – 26	All calculation tasks > Letter matching	ADD
						Mean 24	Calculation with small numbers > Letter matching	ADD
							Approximate addition > Exact addition	ADD
4.	Rickard	2000	5	5	R	Range n/r	Multiplication verification > Control task	MUL
						Mean 24		
5.	Landro	2001	12	n/r	n/r	Range 20 – 45	Add up until 10 (run 2) > Off block	ADD
						Mean 32.5		ADD
6.	Simon	2002	10	7	R	Range 24 – 30	Subtraction > Control	SUB
						Mean 28		
7.	Hanakawa	2003	8	7	n/r	Range 22 – 33	Numeral mental-operation task > Verbal rehearsal task	ADD
						Mean 24		
8.	Molko	2003	14	n/r	R	Range 18 – 30	All calculation tasks > Rest	ADD
						Mean 24.3	Normal exact calculation > Small exact calculation	ADD
							Small approximate calculation > Small exact calculation	ADD
9.	Delazer	2003	13	6	R	Range n/r	Fact retrieval multiplication > Number matching	MUL
							Untrained multiplication > Number matching	MUL
						Mean 30.5	Untrained multiplication > Trained multiplication	MUL
10.	Hanakawa	2003b	16	7	15 R	Range 22 – 34	Mental operation task > Verbal rehearsal task	ADD
						Mean 24		
11.	Kawashima		8	4	R	Range 40 – 49	Addition > Baseline control	ADD
						Mean 44.1	Subtraction > Baseline control	SUB
							Multiplication > Baseline control	MUL
12.	Delazer	2004	13	n/r	R	Range n/r	Untrained multiplication > Trained multiplication	MUL
						Mean n/r		
13.	Hugdahl	2004	12	7	n/r	Range 25 – 36	Mental Arithmetic Task > Vigilance task	ADD
						Mean 31		
14.	Audoin	2005	10	7	R	Range n/r	PASAT task (addition) > Control task	ADD
						Mean 26.6		
15.	Venkatraman	2005	10	3	R	Range 20 – 25	Exact addition symbolic > Control task	ADD
						Mean n/r	Approximate addition symbolic > Control task	ADD
							Exact addition non-symbolic > Control task	ADD
							Approximate addition non-symbolic > Control task	ADD
16.	Kong	2005	16	9	R	Range 25 – 36	Addition with carrying > Control condition	ADD
						Mean 28	Addition without carrying > Control condition	ADD
							Subtraction with borrowing > Control condition	SUB
							Subtraction without borrowing > Control condition	SUB
17.	Wang	2005	18	9	R	Range 22 – 36	Continuous mental arithmetic (subtraction) > Relax	SUB
						Mean n/r	Repeated recitation of multiplication tables > Relax	MUL
18.	Venkatraman	2006	20	7	R	Range n/r	Base−7 Addition before train > Base−7 Addition after train	ADD
						Mean n/r		
19.	Ischebeck	2006	12	8	n/r	Range n/r	Multiplication untrained > Baseline	MUL
						Mean 26.8	Multiplication trained > Baseline	MUL
							Multiplication untrained > Multiplication trained	MUL
							Subtraction untrained > Baseline	SUB
							Subtraction trained > Baseline	SUB
							Subtraction untrained > Subtraction trained	SUB
20.	Fehr	2007	11	6	R	Range n/r	Complex Addition > Simple Addition	ADD
						Mean 26.8	Complex Subtraction > Simple Subtraction	SUB
							Complex Multiplication > Simple Multiplication	MUL
21.	Hugdahl	2007	12	5	R	Range n/r	PASAT task (addition) > Baseline	ADD
						Mean 31		
22.	Sammer	2007	20	10	R	Range 20.3 – 39.6	Add numbers from 0 to 20 > Reference task	ADD
						Mean 25.4		
23.	Zhou	2007	20	10	R	Range 18.3 – 29.8	Addition small > Fixation	ADD
						Mean 22.7	Addition large > Fixation	ADD
							Multiplication small > Fixation	MUL
							Multiplication large > Fixation	MUL
24.	Grabner	2007	25	0	n/r	Range 22 – 32	Multi-digit multiplication > Single- digit multiplication	MUL
						Mean 25.38 and 25.92		
25.	Tan	2007	22	9	R	Range n/r	Numerical computation and size judgment task > Judgment task	SUB
						Mean n/r		
26.	Ischebeck	2007	18	9	R	Range n/r	Multiplication novel > Multiplication repeated	MUL
						Mean 27.8		
27.	Kuo	2008	12	6	R	Range 21 – 29	Single addition > Baseline	ADD
						Mean 25	Dual addition > Baseline	ADD
							Single subtraction > Baseline	SUB
							Dual subtraction > Baseline	SUB
28.	Zago	2008	14	8	R	Range 20 – 27	Number manipulation > Maintenance	ADD
						Mean 23.5		
29.	Ischebeck	2009	17	7	R	Range n/r	Multiplication untrained > Multiplication trained	MUL
						Mean 25		
30.	Jost	2009	18	9	R	Range 22 – 36	Large multiplication > Small multiplication	MUL
						Mean 24.5	Small multiplication > Zero multiplication	MUL
31.	Grabner	2009	28	0	R	Range 22 – 33	Multiplication of Arabic digits > Figural-spatial task	MUL
						Mean 26.9	Multiplication untrained > Multiplication trained	MUL
32.	Harada	2013	24	24	R	Range n/r	Exact calculation > Control	ADD
						Mean 20.38	Approximate Calculation > Control	ADD
							Approximate Calculation > Exact calculation	ADD
33.	Pinel	2013	64	0	R	Range n/r	Subtraction > Control	SUB
						Mean 23.2		
34.	Prado	2013	53	30	R	Range 19 – 30	Multiplication large > Multiplication small	MUL
						Mean 24.2		
35.	Yang	2013	17	7	R	Range n/r	Addition task (AT) > Memory task (MT)	ADD
						Mean 25.76	Subtraction task (ST) > Memory task (MT)	SUB
36.	Bulthe	2014	16	12	1 L	Range 21 – 28	Localizer task (Subtraction) > Fixation	SUB
						Mean n/r		
37.	De Visscher	2015	20	10	R	Range 23 – 34	Multiplication large > Multiplication small	MUL
						Mean 29		
38.	Andin	2015	15	12	R	Range 22 – 37	Multiplication > Visual control	MUL
						Mean 28.6	Multiplication > Cognitive control	MUL
							Subtraction > Visual control	SUB
							Subtraction > Cognitive control	SUB
39.	Kanjlia	2016	19	9	n/r	Range n/r	Math task (subtraction) > Sentences	SUB
						Mean 46		
40.	Bloechle	2016	32	24	R	Range n/r	Multiplication untrained > Multiplication trained (UT2-T)	MUL
						Mean 22		
41.	Soylu	2016	13	6	R	Range 20 – 29.34	Two-digit addition > One-digit addition	ADD
						Mean 24.67		
42.	Pletzer	2016	74	34	R	Range 18 – 40	Two-digit subtraction > Null events	SUB
						Mean ∼ 25.4	Single-digit multiplications > Null events	MUL
43.	Chang	2016	26	13	R	Range 19.0 – 22.6	Complex subtraction > Number Identification	SUB
						Mean 20.6		
44.	Yang	2017	17	n/r	R	Range n/r	Addition task (AT) > Number matching task (NT)	ADD
						Mean 25.76	Subtraction task (ST) > Number matching task (NT)	SUB
45.	De Visscher	2018	42	29	R	Range 18 – 48	Multiplication verification task: large > small	MUL
						Mean 22		
46.	Bugden	2019	24	17	R	Range 18 – 34	Symbolic addition (SA) > Color control (NC)	ADD
						Mean 21.92	Non-symbolic addition (NA) > Color control (NC)	ADD
47.	Matejko	2019	26	12	R	Range 19.5 – 26.3	Addition task: large problem > Plus 1 Problem	ADD
						Mean 22.2	Addition task: small problem> Plus 1 Problem	ADD
48.	Castaldi	2020	16	9	n/r	Range 23 – 27	Calculation task (subtraction) > Reading	SUB
						Mean 25		
49.	Heidekum	2021	46	28	R	Range 19 – 32 and 21 – 35	Multiplication untrained > Multiplication trained	MUL
						Mean 23 and 24		
50.	Matejko		26	12	R	Range 19.5 – 26.3	Addition large problem task > Addition small problem task	ADD
						Mean 22.2		
51.	Amalric	2022	14	n/r	n/r	Range n/r	Forced-choice math task > Math naturalistic video lesson	MUL
						Mean n/r		
52.	Göbel	2022	18	10	R	Range 19 – 37	Subtraction > Control	SUB
						Mean 22.06	Multiplication > Control	MUL

The addition category included the following contrast types: (a) Addition > Control Task, (b) Addition Hard > Addition Easy, (c) Addition > Baseline, (d) Addition > Rest, (e) Addition > Fixation, (f) Addition before train > Addition after train. The Subtraction category included the following contrast types: (a) Subtraction > Control Task, (b) Subtraction Hard > Subtraction Easy, (c) Subtraction > Baseline, (d) Subtraction > Rest, (e) Subtraction > Fixation, (f) Subtraction before train > Subtraction after train. The Multiplication problem maps included the following contrast types: (a) Multiplication > Control Task, (b) Multiplication Hard > Multiplication Easy, (c) Multiplication > Baseline, (d) Multiplication > Rest, (e) Multiplication > Fixation, (f) Multiplication before train > Multiplication after train. The Division category comprised four articles within the adults' group (Fehr et al., 2007, Wood et al., 2008, Ischebeck et al., 2009, Rosenberg-Lee et al., 2011), whereas no articles were available for the children's group.

### 2.3 Activation likelihood estimation (ALE)

We used open-source software GingerALE (version 3.0.2; www.brainmap.org/ale/). ALE is a coordinate-based meta-analytical method that combines data from multiple experiments investigating a common cognitive phenomenon (Turkeltaub et al., 2002, Turkeltaub et al., 2012, Eickhoff et al., 2016, Eickhoff et al., 2017). This method quantifies the probability of continuous activation in a specific voxel across multiple sources within a predefined category or group and enables the examination brain regions associated with distinct functions of interest. To have sufficient power to detect moderate effect sizes categories of interest should have 17 or more experiments (i.e., contrasts; Eickhoff et al., 2017). Because of lack of sufficient experiments, we could not perform meta-analyses on the category of division. To standardize all coordinates to a common coordinate system, the built-in Lancaster transform (icbm2tal) was employed to convert all MNI coordinates to Talairach space. To visualize the results obtained from the ALE maps, we utilized the AFNI software (version AFNI for Mac OS versions 10.9–15; Cox, 1996).

### 2.4 Single dataset analyses

Single dataset ALE meta-analyses were performed for adults for arithmetic operations of addition, subtraction, and multiplication. Single datasets for children, ALE meta-analyses were performed for addition, subtraction, and multiplication. We note that multiplication had 11 experiments, however, we decided to analyze this category as it may be indicative of trends. All single dataset ALE analyses were subjected to a cluster-level correction for controlling multiple comparisons of p = 0.05, utilizing a cluster-forming threshold of p < 0.001 (Eickhoff et al., 2012).

### 2.5 Conjunction and contrast analyses

GingerALE was employed to conduct conjunction and contrast analyses in order to ascertain common and distinct areas across arithmetic operation in adults and children. For these analyses, an uncorrected threshold of p < 0.001 was utilized, with 5000 threshold permutations and a minimum volume criterion of 50 mm^3^, as it was on images that already surpassed the rigorous thresholds of cluster-level correction of p = 0.05 and cluster-forming threshold of p = 0.001 employed in the creation of the single-file maps (Eickhoff et al., 2012). The same threshold parameters were employed for conjunction and contrast analyses conducted between the age groups (i.e., children vs adults) for each arithmetic operation. Notably, GingerALE software allow comparisons (conjunction and contrast) between two datasets at a time, thus we directly compared arithmetic operations within each age groups (e.g., children addition vs children subtraction; adult addition vs adult subtraction) and each arithmetic operation between groups (e.g., addition adults vs addition children).

## 3 Results

Our sample consisted of adults (Addition, n = 376, Males =151, 25.8 ±4.93 years-old; Subtraction, n =424, Males =208, 28.18 ± 6.62 years-old; Multiplication, n =424, Males = 247, 28.18 ± 6.62 years-old) and children (Addition, n = 653, Males = 253, 9.43 ±1.7 years-old; Subtraction, n =268, Males =110, 10.09 ± 1.43 years old; Multiplication, n =266, Males =118, 10.73 ± 0.85 years-old).

### 3.1 Single dataset meta-analyses

**Fig. 2:**
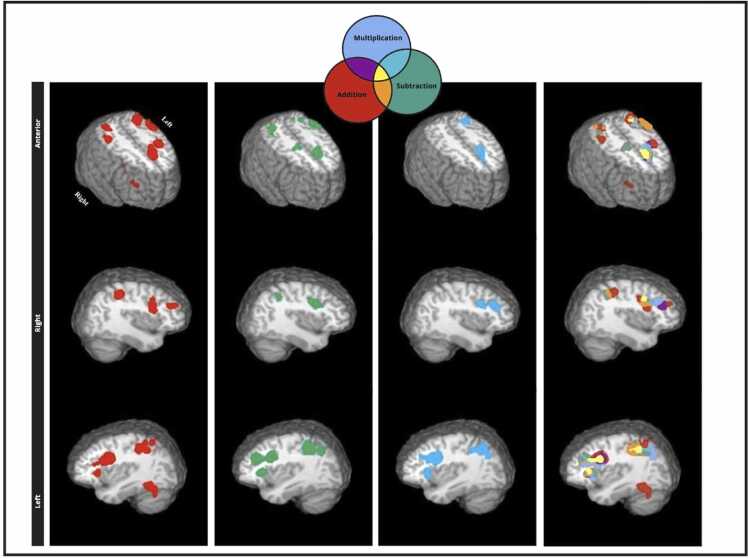
Single dataset ALE maps of addition, subtraction, multiplication, and a visual overlay of all operations (addition, subtraction, multiplications) in adults. These maps were generated in AFNI using a cluster-level correction of 0.05 with 1000 threshold permutations and a cluster-forming threshold of p < 0.001. Coordinates in Talairach space and brain labels are in Table 3.

**Fig. 3:**
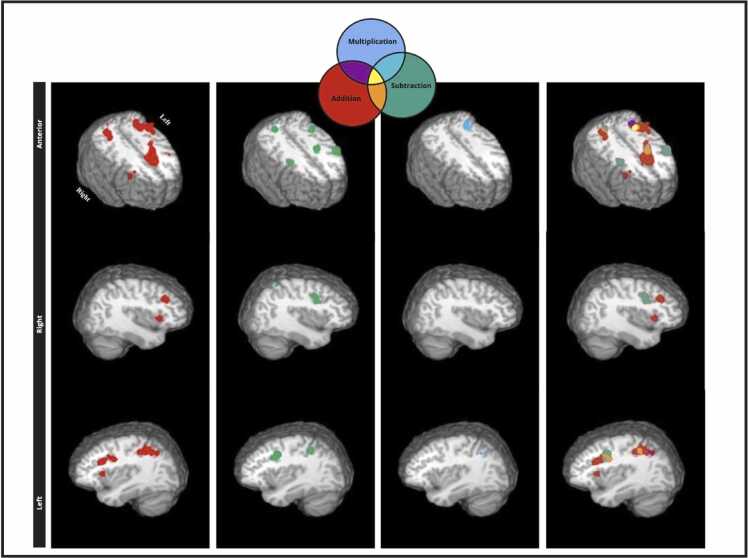
Single dataset ALE maps of addition, subtraction, multiplication, and a visual overlay of all operations (addition, subtraction, multiplications) in children. These maps were generated in AFNI using a cluster-level correction of 0.05 with 1000 threshold permutations and a cluster-forming threshold of p < 0.001. Coordinates in Talairach space are reported in Table 3.

Brain areas concordant across experiments that examine arithmetic operations: addition, subtraction, and multiplication for children and adults are illustrated on Fig. 2, Fig. 3. Table 3 list the corresponding coordinates, brain labels and statistical parameters.

**Table 3:** Single dataset analyses for addition, subtraction, and multiplication in adults and children.

	Hemisphere	Brain area	BA	X	Y	Z	ALE	Vol/mm^3^
	**Adults. Addition**							
1.	L	Precuneus	7	−26	−64	38	0.0425	7868
	L	Superior Parietal Lobule	7	−28	−60	48	0.0335	
	L	Inferior Parietal Lobule	40	−32	−50	38	0.0261	
	L	Inferior Parietal Lobule	40	−44	−40	40	0.0260	
	L	Inferior Parietal Lobule	40	−42	−50	50	0.0160	
2.	L	Inferior Frontal Gyrus	9	−50	10	26	0.0414	4808
	L	Inferior Frontal Gyrus	9	−42	4	30	0.0409	
	L	Middle Frontal Gyrus	46	−40	22	18	0.0147	
3.	R	Superior Parietal Lobule	7	28	−58	42	0.0315	3776
	R	Precuneus	19	30	−68	32	0.0202	
4.	L	Superior Frontal Gyrus	6	−4	10	48	0.0450	3280
5.	L	Fusiform Gyrus	37	−46	−54	−12	0.0269	2360
	L	Posterior Lobe. Tuber.		−38	−62	−28	0.0164	
6.	R	Inferior Frontal Gyrus	9	44	10	28	0.0272	2256
7.	R	Insula	13	30	20	6	0.0445	2024
8.	R	Inferior Parietal Lobule	40	40	−40	42	0.0268	1856
9	L	Sub-lobar. Claustrum		−28	20	10	0.0282	1768
10.	L	Middle Frontal Gyrus	6	−26	−4	50	0.0291	1640
11.	R	Middle Frontal Gyrus	46	42	36	24	0.0224	1216
	R	Middle Frontal Gyrus	46	44	44	26	0.0176	
	**Adults. Subtraction**							
1.	L	Precuneus	7	−28	−66	36	0.0327	8968
	L	Inferior Parietal Lobule	40	−42	−48	48	0.0248	
	L	Inferior Parietal Lobule	40	−42	−44	38	0.0240	
	L	Inferior Parietal Lobule	40	−36	−50	38	0.0238	
	L	Precuneus	7	−14	−70	48	0.0217	
2.	L	Inferior Frontal Gyrus	9	−44	6	30	0.0386	5720
	L	Middle Frontal Gyrus	9	−44	28	28	0.0365	
3.	R	Precuneus	19	30	−62	40	0.0473	5400
	R	Inferior Parietal Lobule	40	36	−48	38	0.0267	
4.	R	Inferior Frontal Gyrus	9	42	6	30	0.0301	2592
	R	Middle Frontal Gyrus	9	40	30	26	0.0253	
5.	L	Insula	13	−30	20	6	0.0282	2104
6.	L	Medial Frontal Gyrus	6	−6	6	52	0.0235	2024
	L	Superior Frontal Gyrus	8	0	16	52	0.0206	
	R	Superior Frontal Gyrus	6	4	12	54	0.0200	
7.	R	Insula	13	30	20	6	0.0370	1992
8.	L	Middle Frontal Gyrus	6	−30	0	56	0.0254	1128
9.	R	Middle Frontal Gyrus	6	28	−4	58	0.0175	688
	R	Precentral Gyrus	6	30	−6	52	0.0161	
	**Adults. Multiplication**							
1.	L	Superior Parietal Lobule	7	−28	−60	44	0.0331	5680
	L	Precuneus	19	−30	−70	30	0.0290	
	L	Superior Parietal Lobule	7	−30	−50	40	0.0225	
2.	L	Inferior Frontal Gyrus	9	−42	4	28	0.0507	4296
	L	Middle Frontal Gyrus	9	−46	26	30	0.0171	
	L	Middle Frontal Gyrus	46	−44	18	24	0.0169	
3.	L	Medial Frontal Gyrus	6	−4	4	50	0.0283	2608
		Medial Frontal Gyrus	6	0	18	46	0.0184	
4.	L	Insula	13	−30	20	6	0.0491	2584
5.	R	Sub-lobar. Claustrum.		28	20	8	0.0322	2304
6.	R	Middle Frontal Gyrus	9	42	28	26	0.0337	1784
7.	R	Inferior Frontal Gyrus	9	46	10	28	0.0222	992
	**Children. Addition**							
1.	L	Medial Frontal Gyrus	6	0	18	44	0.0333	3928
	L	Superior Frontal Gyrus	6	−2	8	52	0.0307	
	L	Medial Frontal Gyrus	6	−6	−4	56	0.0162	
2.	L	Inferior Parietal Lobule	40	−42	−48	42	0.0249	3880
	L	Precuneus	19	−28	−64	42	0.0242	
	L	Precuneus	19	30	−64	38	0.0238	
	L	Inferior Parietal Lobule	40	−32	−54	40	0.0222	
3.	R	Insula	13	30	20	8	0.0409	2704
4.	R	Precuneus	7	28	−64	36	0.0258	2448
	R	Inferior Parietal Lobule	40	34	−46	40	0.0200	
	R	Superior Parietal Lobule	7	30	−54	40	0.0193	
5.	R	Middle Frontal Gyrus	9	46	26	30	0.0260	1216
	R	Middle Frontal Gyrus	9	36	20	30	0.0171	
6.	L	Claustrum		−30	16	6	0.0359	1104
7.	L	Middle Frontal Gyrus	9	−38	16	26	0.0223	768
8.	L	Precentral Gyrus	6	−42	−22	30	0.0192	762
	**Children. Subtraction**							
1.	L	Precentral Gyrus	6	−50	0	38	0.0244	1568
2.	R	Inferior Frontal Gyrus	9	42	4	30	0.0145	960
	R	Precentral Gyrus	6	40	2	36	0.0142	
3.	R	Insula	13	32	16	12	0.0178	856
4.	L	Superior Frontal Gyrus	6	−2	4	54	0.0158	704
5.	R	Superior Parietal Lobule	7	36	−52	54	0.0142	664
6.	L	Inferior Parietal Lobule	40	−36	−46	42	0.0147	592
	**Children. Multiplication**							
1.	L	Precuneus	19	−30	−62	38	0.0141	720
	L	Angular Gyrus	39	−32	−56	36	0.0121	
	L	Superior Parietal Lobule	7	−30	−58	44	0.0095	
2.	R	Cingulate Gyrus	32	6	10	42	0.0146	544
	R	Medial Frontal Gyrus	6	8	8	48	0.0092	

Note: Coordinates are in Talairach space; R= right; L= left; BA = Brodmann Areas; Vol=volume.

### 3.2 Conjunction and contrast meta-analyses

Analyses showing common (i.e., conjunction) and distinct (i.e., contrast) brain areas among arithmetic operations in adults and children are listed on Table 4 and Table 5, respectively.

**Table 4:** Conjunction and contrast analyses of Addition, Subtraction, and Multiplication in adults.

	Hemisphere		Brain area	BA	X		Y	Z	ALE	Vol/mm^3^
	**Addition ∩ Subtraction**									
1.	L	Precuneus		7	−28		−64	36	0.0310	5040
	L	Inferior Parietal Lobule		40	−34		−50	38	0.0227	
	L	Inferior Parietal Lobule		40	−42		−44	40	0.0218	
	L	Inferior Parietal Lobule		40	−42		−50	50	0.0166	
	L	Precuneus		7	−20		−72	42	0.0139	
2.	R	Superior Parietal Lobule		7	28		−58	42	0.0301	2952
	R	Precuneus		7	28		−66	32	0.0197	
3.	L	Inferior Frontal Gyrus		9	−44		4	30	0.0364	2552
	L	Middle Frontal Gyrus		46	−44		18	24	0.0142	
4.	R	Insula		13	30		20	8	0.0282	1744
5.	L	Claustrum			−28		20	8	0.0225	1424
6.	L	Medial Frontal Gyrus		6	−6		6	52	0.0235	1400
	R	Superior Frontal Gyrus		6	2		12	52	0.0181	
7.	R	Inferior Frontal Gyrus		9	44		8	28	0.0253	840
8.	L	Frontal-lobe, Sub-Gyral		6	−28		−2	54	0.0202	352
9.	R	Middle Frontal Gyrus		9	42		32	26	0.0194	256
	**Addition > Subtraction**									
		No suprathreshold clusters								
	**Subtraction > Addition**									
1.	L	Middle Frontal Gyrus		9	−44		25	33	3.5400841	368
	**Addition ∩ Multiplication**									
1.	L	Superior Parietal Lobule		7	−28		−62	42	0.0311	3808
	L	Precuneus		19	−28		−70	32	0.0254	
	L	Superior Parietal Lobule		7	−30		−50	40	0.0225	
	L	Precuneus		19	−28		−68	38	0.0220	
2.	L	Inferior Frontal Gyrus		9	−42		4	30	0.0409	3056
	L	Middle Frontal Gyrus		46	−44		16	24	0.0165	
3.	L	Superior Frontal Gyrus		6	−4		6	50	0.0281	1912
	L	Medial Frontal Gyrus		6	0		18	46	0.0182	
4.	L	Sub-lobar. Claustrum		6	−28		20	10	0.0282	1584
5.	R	Sub-lobar. Claustrum			28		20	8	0.0322	1408
6.	R	Inferior Frontal Gyrus		9	46		10	28	0.0222	824
7.	R	Middle Frontal Gyrus		9	44		32	26	0.0204	408
	**Addition > Multiplication**									
		No suprathreshold clusters								
	**Multiplication > Addition**									
		No suprathreshold clusters								
	**Multiplication ∩ Subtraction**									
1.	L	Precuneus		19	−28		−62	40	0.0258	3632
	L	Precuneus		19	−28		70	32	0.0256	
	L	Inferior Parietal Lobule		40	−32		−52	38	0.0193	
2.	L	Inferior Frontal Gyrus		9	−44		6	30	0.0367	2928
	L	Middle Frontal Gyrus		9	−46		26	30	0.0171	
	L	Middle Frontal Gyrus		46	−44		18	26	0.0156	
3.	L	Insula		13	−30		20	6	0.0382	1680
4.	R	Claustrum			28		20	8	0.0322	1480
5.	L	Medial Frontal Gyrus		6	−6		6	50	0.0233	840
6.	R	Middle Frontal Gyrus		9	40		30	26	0.0253	672
7.	R	Inferior Frontal Gyrus		9	44		10	28	0.0211	464
8.	L	Superior Frontal Gyrus		8	0		18	48	0.0164	80
	**Multiplication > Subtraction**									
		No suprathreshold clusters								
	**Subtraction > Multiplication**									
1.	R	Precuneus		7	27		−63	36	3.7190173	1136
	R	Superior Parietal Lobule		7	28		−64	44	3.5400841	
	R	Angular Gyrus		39	34		−62	32	3.352795	
2.	L	Middle Frontal Gyrus		46	−40		26	24	3.5400841	120
	L	Middle Frontal Gyrus		46		−40	30	24	3.352795	
3.	L	Inferior Parietal Lobule		40		−43	−50	42	3.5400841	104

Note: Coordinates are in Talairach space; R= right; L= left; BA = Brodmann Areas; Vol=volume.

**Table 5:** Conjunction and contrast analyses of Addition, Subtraction, and Multiplication in children.

	Hemisphere	Brain area	BA	X	Y	Z	ALE	Vol/mm^3^
	**Addition ∩ Subtraction**							
1.	R	Insula	13	32	16	10	0.0165	608
2.	L	Superior Frontal Gyrus	6	−2	4	54	0.0158	488
3.	L	Inferior Parietal Lobule	40	−36	−48	42	0.0140	248
4.	L	Inferior Frontal Gyrus	6	−44	2	32	0.0127	88
	**Addition > Subtraction**							
		No suprathreshold clusters						
	**Subtraction > Addition**							
		No suprathreshold clusters						
	**Addition ∩ Multiplication**							
1.	L	Precuneus	19	−30	−62	38	0.0141	632
		Angular Gyrus	39	−32	−56	36	0.0121	376
		Superior Parietal Lobule	7	−30	−58	44	0.0095	
	L	Cingulate Gyrus	32	6	10	42	0.0146	
	**Addition > Multiplication**							
		No suprathreshold clusters						
	**Multiplication > Addition**							
		No suprathreshold clusters						
	**Multiplication ∩ Subtraction**							
1.	L	Inferior Frontal Gyrus	9	−48	8	32	0.0133	168
	**Multiplication > Subtraction**							
		No suprathreshold clusters						
	**Subtraction > Multiplication**							
		No suprathreshold clusters						

Note: Coordinates are in Talairach space; R= right; L= left; BA = Brodmann Areas; Vol=volume.

Analyses showing common and distinct brain areas between adults and children are listed on Table 6. Although the conjunction of children and adults show the highest ALE scores in right insula and the left superior frontal gyrus (BA6; adjacent to the dorsal cingulate), the contrast analysis show that adults implicate more extensive set of areas that include prefrontal and temporal areas.

**Table 6:** Conjunction and contrast analyses: Comparing Addition, Subtraction, Multiplication between children and adults.

	Hemisphere	Brain area	BA	X	Y	Z	ALE	Vol/mm^3^
	**Addition: Children ∩ Adults**							
1.	L	Precuneus	19	−28	−64	42	0.0242	2928
	L	Precuneus	19	−30	−64	38	0.0238	
	L	Superior Parietal Lobule	7	−30	−54	40	0.0212	
	L	Inferior Parietal Lobule	40	−42	−46	42	0.0201	
2.	L	Superior Frontal Gyrus	6	−2	8	52	0.0301	1744
	L	Medial Frontal Gyrus	6	2	−2	16	0.0266	
3.	R	Insula	13	30	20	6	0.0405	1672
4.	R	Superior Parietal Lobule	7	30	−54	40	0.0193	1368
	R	Precuneus	7	28	−62	38	0.0191	
	R	Superior Parietal Lobule	7	28	−66	40	0.0184	
	R	Precuneus	7	28	−66	32	0.0182	
5	L	Claustrum		−28	20	8	0.0282	704
6.	L	Precentral Gyrus	6	−42	−2	30	0.0192	632
7.	L	Middle Frontal Gyrus	9	−42	14	26	0.0165	288
	L	Middle Frontal Gyrus	9	−38	12	28	0.0161	
8.	R	Inferior Parietal Lobule	40	38	−44	40	0.0157	80
	**Addition: Children > Adults**							
		No suprathreshold clusters						
	**Addition: Adults > Children**							
1.	L	Inferior Frontal Gyrus	9	−48	6	24	3.7190173	904
	L	Inferior Frontal Gyrus	44	−54	10	20	3.5400841	
2.	L	Fusiform Gyrus	37	−42	−50	−14	3.2388802	152
	L	Fusiform Gyrus	37	−42	−54	−11	3.0902321	
	**Subtraction: Children ∩ Adults**							
1.	L	Precentral Gyrus	6	−46	4	34	0.0213	912
2.	R	Inferior Frontal Gyrus	9	42	4	30	0.0145	752
	R	Precentral Gyrus	6	42	2	36	0.0141	
3.	R	Insula	13	32	16	12	0.0178	592
4.	L	Inferior Parietal Lobule	40	−36	−46	40	0.0143	376
5.		Medial Frontal Gyrus	6	4	54	6	0.0141	248
6.	R	Superior Parietal Lobule	7	34	−56	50	0.0116	72
	**Subtraction: Children > Adults**							
		No suprathreshold clusters						
	**Subtraction: Adults > Children**							
1.	L	Angular Gyrus	39	−29	−62	37	3.7190173	2504
	L	Precuneus	19	−30	−68	42	3.5400841	
2.	L	Middle Frontal Gyrus	9	−44	27	27	3.7190173	2072
	L	Middle Frontal Gyrus	46	−47	34	24	3.5400841	
	**Multiplication: Children ∩ Adults**							
1.	L	Precuneus	19	−30	−62	38	0.0141	704
		Angular Gyrus	39	−32	−56	36	0.0121	
		Superior Parietal Lobule	7	−30	−58	44	0.0095	
	**Multiplication: Children > Adults**							
		No suprathreshold clusters						
	**Multiplication: Adults > Children**							
		No suprathreshold clusters						

Note: Coordinates are in Talairach space; R= right; L= left; BA = Brodmann Areas; Vol=volume.

## 4 Discussion

Brain coordinates associated with separate arithmetic operations in adults and children were examined using systematic review and quantitative ALE meta-analyses. We highlight three key findings:

(a) Adults show concordance in a widespread set of brain regions when solving arithmetic operations in both hemispheres. Although brain areas are similar among arithmetic operations (e.g., parietal, insular and prefrontal regions) we note that they exhibit varied strength order in terms of ALE scores. For instance, the left superior frontal gyrus (BA 6) and right insula have the highest likelihood of being detected for addition, the right precuneus and left inferior frontal gyrus show the highest likelihood of being detected for subtraction, and for multiplication the top ALE scores are observed in the left inferior frontal gyrus and insula. Conjunction analyses revealed many clusters among arithmetic operations with the highest ALE scores in the left inferior frontal gyrus (BA 9) and parts of the parietal cortex, suggesting that implication of the fronto-parietal network is common for math problem solving in adults. Contrast analyses among operations show few significant differences in favor of subtraction in left middle frontal gyrus (BA 9) (subtraction > addition) and left middle frontal gyrus (BA 46) and bilateral parietal areas (BA 7, 39, 40; subtraction > multiplication); suggestive of the need of increased fronto-parietal resources for subtraction. Results point to the notion that common cognitive resources underlie mathematical problem solving, consistent with theoretical understanding of implementing general cognitive processes of mental attention or working memory (e.g., Pascual-Leone, 1970; Pascual-Leone and Johnson, 2021) and empirical research that points to a fronto-parietal network that also underlies processes of mental attention or working memory that are not specifically numeric (e.g., Owen et al., 2005; Wang et al., 2019; Yaple et al., 2021 for meta-analyses).
(b) Like adults, children also activate a widespread set of regions in both hemispheres when solving arithmetic operations, that include areas in the fronto-parietal and cingulo-opercular networks, albeit areas in the cingulo-opercular network show the highest ALE scores. Specifically, the left insula and right claustrum (an area adjacent to the insula) showed the highest concordance for addition, the right precentral gyrus (BA 6; adjacent to the dorsal cingulate) and the left insula showed the highest concordance for subtraction; multiplication showed the highest concordance in the right cingulate gyrus (BA 32; dorsal cingulate) and left precuneus. Conjunction analyses revealed several statistically common regions among operations with the highest ALE scores in the insular cortex (BA 13), cingulate gyrus (BA 32), and inferior frontal gyrus (BA 9). No statistically significant differences among operations were observed, as expected. These results highlight that the cingulo-opercular network, a network that links the insula with the dorsal cingulate is key for solving arithmetic operations in children.
(c) Conjunction analyses for children and adults show that the left BA 6, and the right insula receive the highest ALE scores among other clusters for both addition and subtraction, and the left precuneus for multiplication. Contrasts analyses between age groups showed no concordance for children over and above that of adults, however, adults showed increased concordance in prefrontal, parietal, and temporal cortices.

Overall, results converge in revealing that multiple brain areas are needed in mathematical cognition, albeit the extent of brain area involvement is in part modulated by age and less by operation. Two key networks observed in our analyses are the cingulo-opercular and the fronto-parietal networks. The fronto-parietal network comprises brain regions primarily involving the lateral prefrontal and posterior parietal cortices, including the dorsolateral prefrontal cortex and the inferior parietal lobule. This network is proposed to be involved in all sorts of cognitive processes such as mental attention, working memory and decision-making (Vincent et al., 2008, Arsalidou et al., 2013, Yaple et al., 2021). The cingulo-opercular network encompasses several interconnected brain regions predominantly in the anterior cingulate cortex the anterior insula and adjacent opercular regions (Dosenbach et al., 2007, Gratton et al., 2017). The dorsal cingulate involvement often extents to adjacent regions including superior and medial frontal gyri (BA 6). The cingulo-opercular areas are associated with various tasks that involve cognitive control (Stocco and Anderson, 2008, Sestieri et al., 2014, Aben et al., 2020, Goodman et al., 2020, Zacharopoulos et al., 2023) including mathematical problem solving (Arsalidou et al., 2018, Pollack and Ashby, 2018). The cingulo-opercular network often activates together with the fronto-parietal network.

Descriptive results from within group contrasts show that areas in the cingulo-opercular network mainly exhibit higher ALE scores (i.e., likelihood of being detected) than the fronto-parietal network in children. Descriptive results from within-group contrasts in adults primarily indicate higher ALE scores in areas of the fronto-parietal network. Statistical contrasts reveal regions of common concordance between children and adults in both fronto-parietal and cingulo-operculum regions. However, adults show significantly greater concordance than children in prefrontal, parietal, and temporal regions, but not cingulo-operculum regions. As the cingulo-opercular network favors affective functions (Servaas et al., 2015, Arsalidou and Pascual-Leone, 2016, Hung et al., 2018, van den Heuvel and de Wit, 2018) and the fronto-parietal network favors cognitive functions (Kaufmann et al., 2011, Qiao et al., 2020), the results seem to be in agreement with the notion that every cognitive action requires an affective motivation (Arsalidou and Pascual-Leone, 2016). In essence, the data suggest that mathematical problem solving requires both cognitive and affective processes for both children and adults, albeit adults show statistically more concordance in areas that support cognitive processes. We highlight empirical and theoretical links with past literature. We also provide maps in stereotaxic space for adults and children that can benefit future studies that examine addition, subtraction, and multiplication.

Arithmetic operations in adults engage a widespread set of areas in the fronto-parietal and cingulo-opercular networks, in agreement past meta-analyses (Arsalidou and Taylor, 2011, Pollack and Ashby, 2018, Hawes et al., 2019, Sokolowski et al., 2023). Although we did not except significant differences among arithmetic operations, we identified increased concordance for subtraction tasks for adults that show heightened engagement of the right inferior frontal gyrus and the left superior parietal cortex. The right inferior frontal gyrus is recognized for its role in inhibitory control (Cai et al., 2014, Sebastian et al., 2016, Schroeder et al., 2020, Boen et al., 2022, Choo et al., 2022, Brown et al., 2023) and cognitive flexibility (Marklund and Persson, 2012, Rudebeck et al., 2013, Schaum et al., 2021, Sundby et al., 2021). As part of the lateral fronto-parietal network these areas play a key role in core cognitive resources that support mental attention (Arsalidou et al., 2013, Arsalidou and Pascual-Leone, 2016, Pascual-Leone and Johnson, 2021) and working memory (Rottschy et al., 2012, Yaple et al., 2021 for metanalyses). The execution of subtractive operations typically requires regrouping and borrowing of numbers that may correspond to this increased involvement. Others have discussed unique cognitive demands and strategies intrinsic to each operation (Grabner and De Smedt, 2011). Addition and multiplication were proposed to be of automatic nature. Automatic calculations predominantly rely on rote memorization and factual retrieval, potentially accounting for the comparable brain activation patterns between addition and multiplication. Some also consider that math problem solving skills rely on core resources that change with development (Agostino et al., 2010, Arsalidou et al., 2018). Compared to addition and multiplication, subtraction entails more intricate processes, such as regrouping and borrowing, necessitating heightened cognitive control, and working memory (Campbell and Xue, 2001, Campbell, 2008). We did not anticipate differences among arithmetic operations because we assumed comparable cognitive demands among addition, subtraction, multiplication. However, subtraction problems in adults may have been more demanding in terms of processing steps, thus, the distinct brain areas observed for subtraction may reflect distinct cognitive demands associated with this operation in adults.

For children, all arithmetic operations engage brain areas in fronto-parietal and cingulo-opercular networks, albeit the cinculo-opercular areas generally show higher likelihood of being detected. Specifically, the insula (BA 13), claustrum, superior frontal gyrus (BA 6), and cingulate gyrus (BA 32) were among the regions with the highest ALE scores. These areas were also evident in the conjunction analyses among operations. The anterior insula is located deep within the lateral fissure and has been related with all sorts of affective and cognitive processes (Menon and Uddin, 2010, Duerden et al., 2013, Gasquoine, 2014, Uddin et al., 2017, Schimmelpfennig et al., 2023). The claustrum is a thin strip of cortex adjacent to the insula and is proposed to have a multimodal integrative role (Bennett and Baird, 2006) and as a primary region associated with consciousness (Goll et al., 2015). In mathematical cognition the insula was proposed to be a toggler between goal-directed and default-mode processes and the claustrum to have a role in motivated top-down processes (Arsalidou et al., 2018). As most neuroimaging studies analyze brain correlates of individuals who actively engage in problem solving with satisfactory performance, the rationale behind this explanation becomes apparent. The superior frontal gyrus (BA 6) is adjacent to the dorsal cingulate gyrus and often activates in various studies of core cognitive processes such as working memory (Rottschy et al., 2012, Yaple et al., 2021 for meta-analyses), inhibitory control (Hung et al., 2018, Li et al., 2022 for meta-analyses), and cognitive flexibility (Armbruster et al., 2012, Chen et al., 2014, Wendiggensen and Beste, 2023). In mathematical cognition, the role of the dorsal cingulate has been linked to implementing cognitive goals (Arsalidou et al., 2018). This is consistent with the rational that children who do not adhere to their cognitive goal and solve the math problem correctly, their data are likely to be eliminated from analyses because the researchers cannot directly determine whether the response was given because the child was unable to do the task or unmotivated to do the task. Therefore, as part of the cingulo-opercular network, the insula and the dorsal cingulate, we propose that they have a generic role that underlies mechanisms related motivated task engagement.

The lateral fronto-parietal network has been discussed extensively as part of the working memory network (Yaple et al., 2019, Emch et al., 2019 for meta-analyses) and other executive functions (Houdé et al., 2010, Desco et al., 2011, Ardila et al., 2017). In mathematics the role of fronto-parietal regions has been associated with the procedural manipulation of numerical quantities (Prado et al., 2014, Van Der Auwera et al., 2023). Specifically, reports suggest that the angular gyrus, supports the ability to retrieve arithmetic facts in both typically developing children and adults (Sokolowski et al., 2023b). In number and calculation tasks, the inferior and middle frontal gyri have been associated with monitoring simple rules and more complex rules, respectively (Arsalidou et al., 2018).

Notably, as expected, we did not observe statistically significant differences among arithmetic operations in children. Considering that children included in the articles we analyzed were 9 and 10 years old on average for addition and subtraction and closer to 11 years old for multiplication, one could suggest that children’s abilities in arithmetic operations greatly improve through learning during early school years (Entwisle and Alexander, 1989, Clements and Sarama, 2007, Rodic et al., 2018) and variability in brain response may be expressed in less detectable differences. The lack of differences in brain correlates among arithmetic operations may be also due to comparable experience across operation types (Evans et al., 2016, Brignoni-Pérez et al., 2021) as 9–10-year-olds should have had a couple of years of experience in addition and subtraction and 11-year-olds should have a couple of years of experience in multiplication. This is consistent with the theoretical proposal that children can comfortably solve arithmetic operations within the limits of their mental attentional capacity (Agostino et al., 2010; Pascual-Leone et al., 2010) and our analyses demonstrates the brain correlates of children that can inform this theoretical idea.

Compared to children, adults with an average age of about 27 years old have more extensive experience with all basic mathematical operations. Although conjunction analyses between children and adults shows common fronto-parietal and cingulo-opercular regions, confirming single dataset analyses, studies with adults showed statistically more concordance in prefrontal, parietal, and temporal regions than children. Specifically, contrasts between adults and children showed significant increase in concordance in the inferior frontal gyrus (BA 9, 44) and fusiform gyrus (BA 37) in the left hemisphere for addition, and left hemisphere angular gyrus (BA 39), precuneus (BA 19) and middle frontal gyrus (BA 9/46) for subtraction. Children did not show any concordance above and beyond brain areas implicated in math problem solving in adults. The inferior frontal gyrus, particularly BA 44 is acknowledged to be intricately associated with the region commonly referred to as the Broca area (Kulik et al., 2022). Broca's area retains its centrality in language processing; however, its role transcends mere language production, encompassing higher-order cognitive functions that pertain to working memory (Chein et al., 2002). The left fusiform gyrus often assumes a significant role in the recognition and identification of alphabetical strings, facilitating their transmission to regions implicated in number processing and retrieval of arithmetic facts (Venkatraman et al., 2006). The left angular gyrus, whose spatial localization is associated with Wernicke's area known for its role in language comprehension (Binder, 2017, Jäncke et al., 2021), demonstrated an increased activation correlating with chronological age (Rivera et al., 2005). The observed outcome could potentially be attributed to the prevalence of language-based strategies employed by adults when engaging in mathematical problem-solving tasks (Kwon et al., 2002). Therefore, the activation differences between adults and children could reflect developmental changes in the organization and functionality of these brain regions over time. Moreover, the findings may suggest that certain cognitive strategies or approaches used by adults in the tasks are not yet fully established in children. Overall, concordance in these brain regions in adults and their absence in children provides valuable insights into the neural underpinnings of cognitive and mathematical processing during the analyzed tasks, highlighting the developmental differences between these two groups in terms of brain function and cognitive abilities.

## 5 Limitations

We note that fMRI meta-analyses in general cannot control for variability in statistical methodologies and thresholding approaches used by original studies. The present meta-analyses used peak coordinates reported by original peer-reviewed published studies that examine arithmetic operations in children and adults. We note two considerations for interpreting the results of the present meta-analyses. First, we did not control for task difficulty within each arithmetic operation which may have affected the participants’ computational strategies, however, most articles used single digit numbers for addition and subtraction, and most articles use problems in the multiplication table. Second, we point out again that we only had 11 experiments with multiplication problems in children rather than a minimum of 17 experiments (Eickhoff et al., 2017), thus results related to children’s multiplication tasks is underpowered.

## 6 Conclusion

We conducted a comprehensive investigation into brain activations associated with separate mathematical operations in both children and adults. We identified several key findings that contribute to our understanding of the neural underpinnings of mathematical processing. Our within-group results revealed that children and adults show concordance in frontal-parietal and cingulo-opercular areas with children generally showing higher ALE scores in the later. Subtraction elicits significantly distinct concordance mainly of fronto-parietal regions, in adults, but not in children. Division remains an understudied operation and further research is needed. Theoretically, our results are consistent with general, constructive theories of cognitive development (e.g., Pascual-Leone, 1970; Pascual-Leone and Johnson, 2021). The stereotaxic coordinate maps we provide serve as a benchmark for future research with neurodevelopmental disorders as well as for individuals exhibiting exceptional mathematical proficiency.

## Funding

This article is the output of a research project implemented as a part of the Basic Research Program at the 10.13039/501100007251National Research University Higher School of Economics (HSE University). Author AI has received research support from The Brain Program of the IDEAS Research Center.

## CRediT authorship contribution statement

**Asya Istomina:** Writing – original draft, Visualization, Formal analysis, Conceptualization. **Marie Arsalidou:** Writing – review & editing, Supervision, Conceptualization.

## Declaration of Competing Interest

The authors declare that they have no known competing financial interests or personal relationships that could have appeared to influence the work reported in this paper.
